# Editorial: Isotopic labeling approaches for exploring plant metabolism dynamics

**DOI:** 10.3389/fpls.2023.1356153

**Published:** 2024-01-08

**Authors:** Younès Dellero, Zoran Nikoloski

**Affiliations:** ^1^ Institut Agro Rennes Angers (INRAE), Université Rennes, Institut Agro, IGEPP-UMR1349, P2M2-MetaboHUB, Le Rheu, France; ^2^ Bioinformatics Department, Institute of Biochemistry and Biology, University of Potsdam, Potsdam, Germany; ^3^ Systems Biology and Mathematical Modeling, Max Planck Institute of Molecular Plant Physiology, Potsdam, Germany

**Keywords:** metabolic flux analysis (MFA), isotopic labeling, 13C discrimination, microalgae, crops

In the context of climate change, understanding plant metabolism - the basis of biomass and defense response - is at the core of next-generation breeding strategies to obtain climate-resilient crops. Indeed, metabolic fluxes are the result of regulation at multiple levels from transcriptional and (post) translational to metabolic regulation. Metabolic fluxes represent the most direct link with agronomically relevant traits, including: growth, tolerance and resistance to (a) biotic stresses, and yield. Hence, it is important to understand how particular metabolic fluxes shape these traits and their response to different environmental cues. These two problems can be addressed by: (*i*) tracing the fate of metabolic probes within the metabolic network and (*ii*) using the resulting data to quantify the activity of individual metabolic reactions and pathways via metabolic flux analysis (MFA). The latter requires dynamic analyses, conducted at isotopically non-stationary state, to obtain informative data. Coupled with analyses of differential flux behavior, such approaches could introduce new targets for improving crop resilience.

Despite the unprecedented production of data from ^13^C-labeling experiments and the recent emergence of dedicated software, ^13^C-INST-MFA remains marginally applied to photosynthesizing organisms. These discrepancies can reflect the complexity of this modelling approach, *i.e.* fitting both metabolite pools and reaction fluxes, dealing with multiple compartments but also its sensitivity to errors of isotopic measurements. In this Research Topic, four studies addressed these methodological bottlenecks by using approaches for the field of plant science. Huß and Nikoloski survey the literature on local approaches for INST-MFA. They systematically compared existing local approaches with respect to the required data and underlying computational problems solved on a synthetic network example. The performance of these approaches in estimating fluxes was benchmarked using a sub-network of *Arabidopsis thaliana* nitrogen metabolism. Important recommendations for isotopic measurements (resolution, accuracy) and metabolic sub-network selection were formulated to maximize flux estimation performances with local flux estimation approaches. Dellero at al. provided an update of ^13^C distribution measurement accuracies by GC-MS using tailor-made ^13^C-standards that had a predictable ^13^C distribution. Using the validated MS method and U-^13^C-pyruvate incorporations into *Brassica napus*, they used the pathway fractional contribution (local approach) to compare phosphoenolpyruvate carboxylase and tricarboxylic acid (TCA) cycle fluxes. They also identified a cyclic mode of the TCA cycle in the light and questioned the contribution of stored citrate to this cycle. Küken et al. proposed a new and simplified local approach as an alternative to classical INST-MFA. This approach, called simulation-free constrained regression approach (SFCR), formulated flux estimation for Calvin-Benson cycle as a single constrained regression problem, avoiding the need for repeated simulation of time-resolved labeling patterns. Using ^13^C-data from three different microalgae, SFCR approach provided a suitable alternative for flux estimation in data-rich scenarios when compared to INCA tool. Smith et al. addressed the challenge of applying INST-MFA to heterotrophic cells following an oxidative load using U-^13^C-glucose incorporations. INCA-based modelling yielded statistically acceptable flux solutions by tuning different parameters (inactive pools, distinct compartments, remobilization of unlabeled carbon, uncertainty of isotopic measurements) at the cost of precision in flux estimates. Overall, the oxidative load caused only relatively subtle changes in flux through the network of central carbon metabolism.

Besides these cellular functional analyses, two other studies used stable isotopes to investigate important nutritional and physiological processes at the whole-plant level. Yan et al. studied the contribution of complex organic fertilizers to the Zn nutrition of Ryegrass using ^67^Zn-labeled soil. Nearly 83% of Zn was derived from the soil, 15% from the fertilizers and 2% from the seed. The results indicated that most of the freshly added Zn remained in the soil after one crop cycle and may thereby contribute to a residual Zn pool in the soil. Kunz et al. evaluated the suitability of ^13^C discrimination, relative leaf water content and plant canopy temperature to phenotype drought/heat resistance of winter wheat for future climate resilience. Using field trials with different wheat varieties (regionally adapted and German varieties), they concluded that ^13^C discrimination was the best indicator for drought resistance. Given the indirect link between ^13^C discrimination and leaf water content, such results highlighted the central role of leaf metabolism in tolerance to drought.

This Research Topic provides an update of methods and tools to improve both the accuracy of MS-based isotopic measurements and the feasibility of flux estimation in photosynthesizing organisms. The development of local approaches will certainly contribute to increase the application of ^13^C-MFA for the next years in order to easily: (*i*) test specific hypotheses about the activity of particular metabolic pathways in plants and other eukaryotic photosynthesizing organisms; (*ii*) quantify and compare reaction fluxes under different growth scenarios. Given the ability of plant metabolism to influence physiological processes at the whole-plant level, plant fluxomics is an interesting area to investigate to raise targets for developing next-generation climate-resilient crops. While isotopic labeling and ^13^C-MFA approaches are routinely used to describe how cancer cells function, there is still a long way to go before they are routinely used in plants. The development of national and international structures and collaborations in the field of plant fluxomics will definitely speed things up.

## Author contributions

YD: Writing – original draft. ZN: Writing – review & editing.

